# Supplementary material for: Different Cell Types Affect the Transition from Juvenile to Mature Phase in Citrus Plants Regenerated through Somatic Embryogenesis
Source: Plants (Basel). 2022 Jul 8;11(14):1811. doi: 10.3390/plants11141811 (PMC9323018; doi:10.3390/plants11141811)
Supplement: Supplementary file 1 [file plants-11-01811-s001.zip › plants-1742289-supplementary.pdf]

# Different cell types affect the transition from juvenile to mature phase in Citrus plants regenerated trough somatic embryogenesis

**Caterina Catalano[1†], Loredana Abbate[1†], Sergio Fatta Del Bosco[1], Antonio Motisi[1], Francesco Carimi[1*], Roberto De Michele[1], Francesco Mercati[1], Anna Maria D'Onghia[2] and Angela Carra[1]**

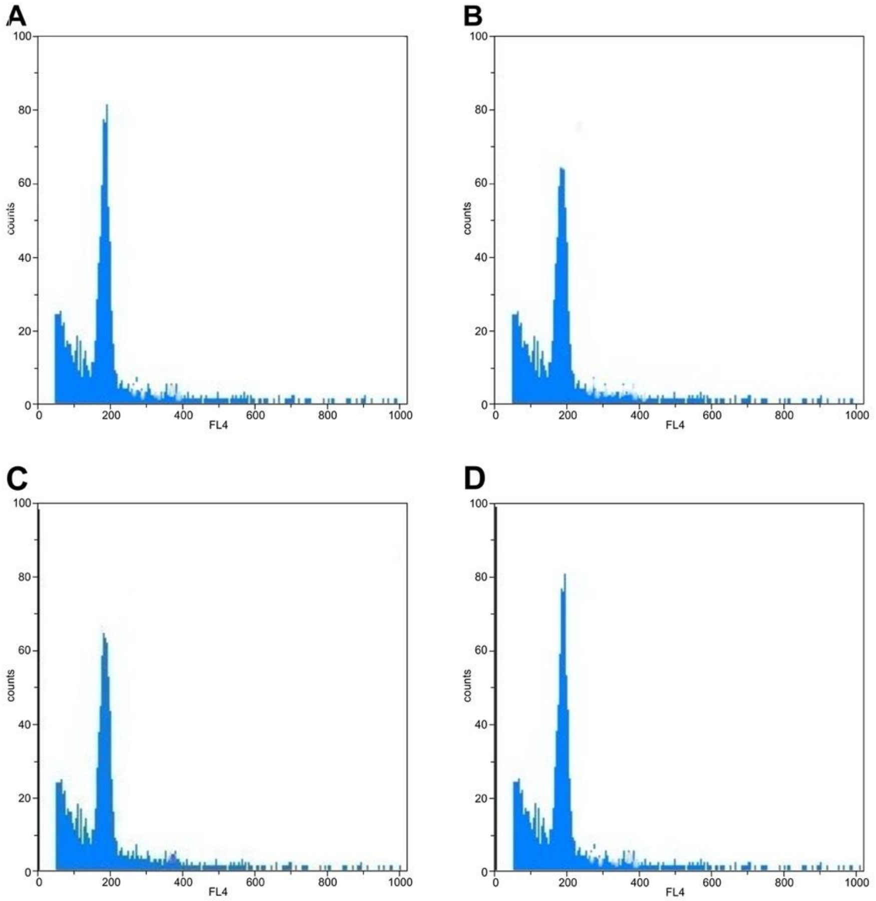


**Figure S1.** Flow cytometry and ploidy evaluation of regenerants of *Citrus limon* 'Lunario' (**A**), *Citrus deliciosa* 'Tardivo di Ciaculli' (**B**), *Citrus sinensis* 'Valencia late' (**C**), *Citrus aurantium* 'AA CNR 31' (**D**). Leaf nuclei suspensions were stained with DAPI. Each sample was analysed using the leaf nuclei of the relative mother plant as internal diploid standard (STD 2C). Nuclei DNA fluorescence intensity values and nuclei counts are shown on X and Y axes, respectively.

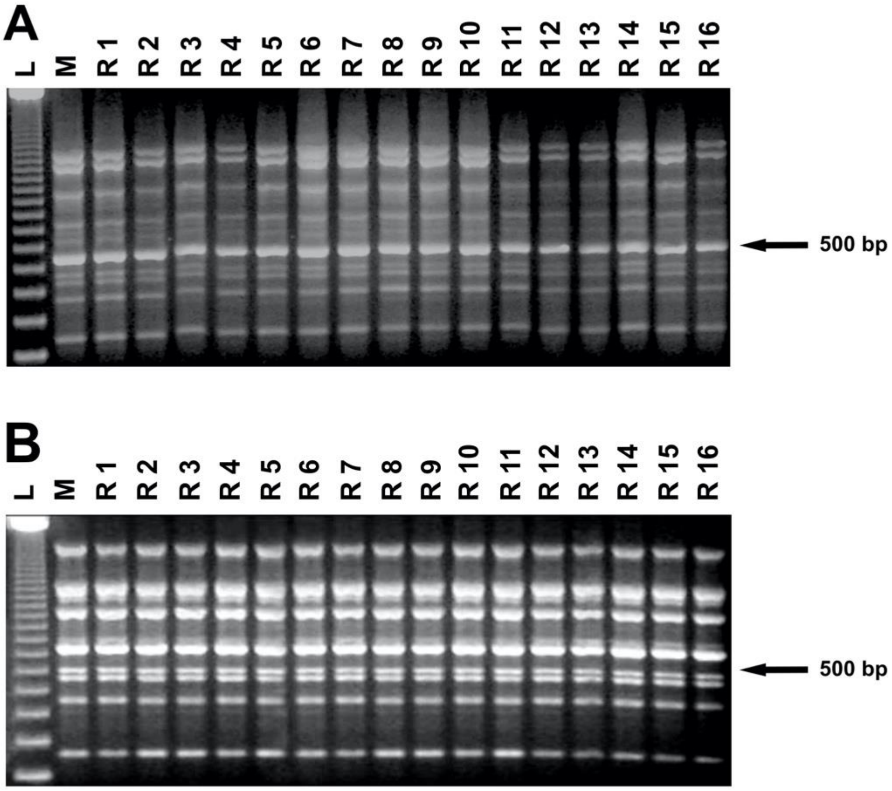


**Figure S2.** Representative images of molecular markers. (**A**) Patterns of mother plant (M) and 16 regenerated plantlets (R1-16) of *Citrus deliciosa* 'Tardivo di Ciaculli' obtained with the ISSR primer UBC-835. L, 100 bp ladder; (**B**) Patterns of mother plant (M) and 16 regenerated plantlets (R1-16) of *Citrus sinensis* 'Valencia late' obtained with the RAPD primer OPM04. L, 100 bp ladder.

**Supplemental Material**

**Supplemental Table S1**. Primer sequences and annealing temperatures used for ISSR analysis.

| Primer Name | Primer Sequence | Annealing Temperature (°C) |
|---|---|---|
| UBC-848 | 5′-(CA)$_8$RG-3′ | 51.0 |
| UBC-857 | 5′-(AC)$_8$YG-3′ | 50.0 |
| UBC-835 | 5′-(AG)$_8$YC-3′ | 52.6 |
| UBC-834 | 5′-(AG)$_8$YT-3′ | 50.3 |
| ISSR4+4b | 5′-(AC)$_8$YA-3′ | 49.0 |
| ISSR10+10b | 5′-(TCC)$_5$RY-3′ | 56.0 |

**Supplemental Table S2.** Primer sequences used for RAPD analysis.

| Primer Name | Primer Sequence |
|---|---|
| OPAT 14 | 5′-GTGCCGCACT-3′ |
| OPH 04 | 5′-GGAAGTCGCC-3′ |
| OPH 15 | 5′-AATGGCGCAG-3′ |
| OPM 04 | 5′-GGCGGTTGTC-3′ |
| OPN 14 | 5′-TCGTGCGGGT-3′ |
| OPO 14 | 5′-GGACGCTTCC-3′ |
